# Synthesis of block copolymer with *cis*-1,4-polybutadiene and isotactic-rich polystyrene using α-diimine nickel catalysts

**DOI:** 10.1098/rsos.230791

**Published:** 2023-10-18

**Authors:** Nan Zheng, Jie Liu, Zonglin Li, Jiufu Lu, Yan Ni, Xin Min

**Affiliations:** ^1^ Shaanxi Key Laboratory of Catalysis, College of Chemical and Environmental Science, Shaanxi University of Technology, Hanzhong, Shaanxi 723001, People's Republic of China; ^2^ Yuzhang Normal University, Nanchang, Jiangxi 330103, People's Republic of China; ^3^ College of Chemistry and Environmental Engineering, Jiujiang University, Jiujiang, Jiangxi 332005, People's Republic of China

**Keywords:** α-diimine nickel, *cis-*1,4 unit, isotactic, di-block, styrene, butadiene

## Abstract

A series of styrene-butadiene di-block copolymers with high *cis*-1,4 unit content (greater than 92%) polybutadiene (PB) and isotactic-rich polystyrene (PS) (*mmmm* > 65%) was synthesized using α-diimine nickel catalysts (Ni-diimine). Four different Ni-diimine catalysts were synthesized via a complexing reaction between nickel (II) naphthenate and laboratory-made α-diimine ligands L1, L2, L3 and L4, which have different steric volume structures. The results indicate that the Ni-diimine catalyst prepared using the L4 ligand with a higher steric volume can effectively initiate the block polymerization of butadiene and styrene, and the resulting polymer has distinguished *cis*-1,4 structure unit PB and high isotactic-selective PS block. Differential scanning calorimetry and electrochemical performance tests show that these block copolymers with *cis*-1,4-regulated and isotactic-selective polymerization have advantages in terms of high-temperature and low-temperature resistance as well as corrosion resistance. Therefore, these copolymers are expected to be widely used in some harsh industrial environments.

## 1. Introduction

Styrene-butadiene block or styrene-butadiene-styrene tri-block copolymers (PS-*b*-PB or SBS) are important thermoplastic elastomers in various industrial fields owing to their excellent properties [1–5]. Many literature reports have shown that the low-temperature resistance and mechanism performance of butadiene rubber or styrene-butadiene block copolymers can be significantly improved by increasing the *cis*-1,4 structure unit content in the butadiene block [6–8]. Meanwhile, the stereoselectivity of polystyrene (PS) block also significantly influences the combined properties of these copolymers, and a high tacticity degree (including isotactic and syndiotactic degree) results in a higher melting point, high tensile modulus, excellent chemical resistance and other desirable properties [9,10].

At present, the methods for synthesizing styrene-butadiene block copolymers include anionic polymerization [11], ring-opening metathesis polymerization (ROMP) [12], and ternary rare earth-catalysed coordination polymerization [13], etc. Among them, the polymers obtained by anionic polymerization and ROMP have *cis*-1,4 structure content of less than 50% [9]. In ternary rare earth-catalysed coordination polymerization, neodymium carboxylate and neodymium phosphonate are usually used as the main catalysts [14,15], and high *cis*-1,4-selectivity (greater than 96%) can be obtained. However, the styrene block in the resulting copolymers generally exhibit atactic sequences, and the monomer conversion rate is very low [13,16].

In recent years, a series of metallocene rare earth catalysts has been applied in the preparation of styrene-butadiene block copolymers by many scholars [3,17]. Cui *et al*. [18] and Hou *et al*. [19], respectively, used a linked-half-sandwich lutetium-bis(allyl) complex and half-sandwich metallocene scandium catalysts to efficiently synthesize styrene-butadiene block copolymers with high *cis*-1,4 unit content and pure syndiotactic selectivity. However, the high cost of the co-catalyst [Ph_3_C][B(C_6_F_5_)_4_] has seriously limited their application. Titanocene and methylaluminoxane catalyst systems can also be used in the synthesis of styrene-butadiene block copolymers. The resulting copolymers exhibit syndiotactic styrene content of 95%, and the *cis*-1,4 structure content of the butadiene is higher than 70%. However, the conversion rate of monomer styrene and butadiene is only about 20% [20].

**Scheme 1.**
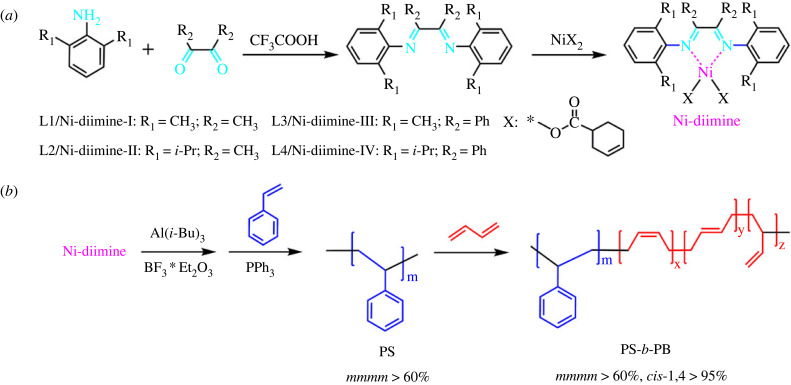
(*a*) Synthesis route of Ni-diimine; (*b*) synthesis route of PS-*b*-PB via Ni-diimine catalysts.

In this article, α-diimine nickel complexes with different structures were synthesized and combined with alkyl aluminium, boron trifluoride ether and triphenyl phosphine to form a catalytic system. This catalytic system was applied to the synthesis of styrene-butadiene block copolymers. Block copolymers with high *cis*-1,4 structure content and rich isotactic styrene block copolymers were prepared. Moreover, the influence of the ligand structure on catalytic activity and the spatial structure of the polymer was studied. The structure of α-diimine and the synthesis process of the copolymer are shown in scheme 1.

## 2. Experimental

### 2.1. Materials

1,3-Butadiene (Bd, 1.9 M in hexane) in a ChemSeal bottle was purchased from Energy China and used as received. Styrene (St, 99%) was obtained from Macklin China. The styrene was purified by distillation over calcium hydride with dilution to 2 M in hexane. 2,3-Butanedione (98%), benzil (98%), 2,6-dimethylaniline (99%), 2,6-diisopropylaniline (96%) and diisobutylaluminium hydride (Al(*i*-Bu)_3_, 1 M in n-hexane) were obtained from Energy China. Boron trifluoride etherate (B, 98%), trifluoroacetic acid (TFA, 99%), triphenyl phosphine (PPh_3_, 97%) and 2,6-di-tert-butyl-p-cresol (Antiager 264, 97%) were purchased from Macklin China and used as received. Nickel (II) naphthenate (Ni, 5 wt %) was supplied by Meryer China and diluted to 0.025 M with cyclohexane. Toluene, cyclohexane (analytically pure reagent (AR)), hexane (AR), methanol (AR), ethanol (AR) and butanone were purchased from Sinopharm Chemical Reagent Co., Ltd. and used as received.

### 2.2. Synthesis of α-diimine ligands

The synthesis routes of the α-diimine ligands are schematically illustrated in scheme 1 [21]. A detailed synthesis procedure of ligand L4 is described as a typical example. First, 5.26 g (25 mmol) benzil, 8.86 g (50 mmol) 2,6-diisopropylaniline, 0.46 g trifluoroacetic acid and 40 ml toluene were added to a 100 ml round-bottom flask. This mixture was heated to 130°C and stirred for 24 h. Then, the solution was transferred to a low-temperature environment of −15°C, and brown-yellow crystals were precipitated. The resulting product was washed with ice methanol and dried under vacuum at 40°C until a constant-weight product was obtained. The obtained solid yellow product was denoted ligand L4. The process yield was 95.2%. ^1^H nuclear magnetic resonance (NMR) (600 MHz, CDCl_3_): *δ* 7.3–8.17(m, 5H), *δ* 6.91–7.22(m, 3H), *δ* 2.90(m, 1H), *δ* 1.07(m, 12H). A series of ligands (L1, L2, L3 and L4, see scheme 1 and table 2 for more detail) was synthesized with the same synthesis process. The detailed synthesis procedures of L1, L2 and L3 are shown in the electronic supplementary material, S1. L1 : ^1^H NMR(600 MHz, CDCl3): *δ* 7.07(d, 2H), *δ* 6.94(t, 1H), *δ* 2.04(d, 9H)–L2: ^1^H NMR(600 MHz, CDCl_3_): *δ* 7.16(d, 2H), *δ* 7.09(m, 1H), *δ* 2.71(m, 2H), *δ* 2.07(s, 3H), *δ* 1.18(m, 12H); L3: ^1^H NMR(600 MHz, CDCl_3_): *δ* 7.16(d, 2H), *δ* 7.09(m, 1H), *δ* 2.71(m, 2H), *δ* 2.07(s, 3H), *δ* 1.18(m, 12H).

### 2.3. Synthesis of α-diimine nickel catalysts

The synthesis routes of the α-diimine nickel catalysts (Ni-diimine-I, Ni-diimine-II, Ni-diimine-III and Ni-diimine-IV) are shown in scheme 1. A detailed synthesis procedure of Ni-diimine-IV is provided as a typical example. First, 2.64 g (5 mmol) L4, 5.87 g (5 mmol) nickel naphthenate and 38 ml cyclohexane were added to a 100 ml round-bottom flask. The reaction was carried out at room temperature for 3 h to obtain a green transparent solution. Next, the catalyst solution was transferred to a constant-temperature environment at 5°C for static storage until yellow-green crystals were precipitated. After filtration, the crystals were transferred to an oven at 70°C and dried to a constant weight. Finally, the resulting product was dissolved in cyclohexane to form a stable complex solution with a concentration of 0.025 M.

### 2.4. Synthesis of styrene-butadiene di-block copolymer using α-diimine nickel catalysts

All synthesis operations were conducted under a dry argon atmosphere. The detailed polymerization procedure of PS-*b*-PB-2 (table 1) is described as a typical example. Ni-diimine-IV solution (Ni, 0.01 mmol, 0.1 M in cyclohexane), styrene (St, 0.25 mmol, 2 M in cyclohexane), Al(*i*-Bu)_3_ (Al, 0.2 mmol, 1 M in *n*-hexane) and boron trifluoride etherate (B, 0.3 mmol, [B]/[Al] = 1.5) were sequentially injected into a Schlenk tube with a rubber septum by a syringe. This mixture was aged at 50°C under continuous stirring. After 15 min, triphenyl phosphine (PPh_3_, 0.01 mmol) was injected, and the mixture was aged for another 15 min. After ageing, a reddish brown transparent catalyst solution was obtained.

**Table 1.** Synthesis of PS-*b*-PB via Ni-diimine-IV^a^.

entry	monomer	conv^b^ (%)	St cont^c^ (%)	*M*_n_^d^ × 10^4^ g mol^−1^	*M*_w_/*M*_n_^d^	*cis*-1,4^e^ (%)	*mmmm*^f^ (%)
1	St	96.5	100	4.3	1.32	—	65.2
2	Bd	82.1	29.9	13.8	1.59	95.2	65.3

^a^1, 2 correspond to the first, second polymerization steps. First step, [Ni] = 0.01 mmol, Catalyst ratio: [Ni]/[St]/[Al]/[B]/[PPh_3_] = 1 : 10 : 20 : 30 : 1, [St]/[Ni] = 400, second step, [Bd]/[Ni] = 2000.

^b^Conversion of monomer in each polymerization steps.

^c^Determined by ^1^H NMR.

^d^Determined by SEC-MALLS.

^e^Determined by ^1^H NMR and ^13^C NMR.

^f^Determined by ^13^C NMR.

Next, the styrene solution (2 mmol, 2 M in hexane, [St]/[Ni] = 400) was injected into the Schlenk tube with the preformed catalyst solution using a syringe. Polymerization was carried out at 50°C for 3 h. Then, butadiene solution (Bd, 10 mmol, 1.9 M in *n*-hexane, [Bd]/[Nd] = 2000) was added. This polymerization was carried out at 50°C for 3 h under stirring, followed by quenching with ethanol containing antiager 264 (1 wt %) as a stabilizer. The resulting product was precipitated in ethanol and repeatedly washed with ethanol, then extracted with butanone and *n*-hexane three times each. Finally, the product was dried under vacuum at 55°C until a white solid with a constant weight was obtained.

### 2.5. Characterization

The Fourier transform-infrared (FT-IR) spectra of the α-diimine ligands and resulting polymers were measured by a Bruker VERTEX70 spectrophotometer. ^1^H NMR and ^13^C NMR spectra were recorded by a Bruker 400 MHz instrument using CDCl_3_ as the solvent. For the polybutadiene (PB) and PS-*b*-PB polymers, the ratio of 1,4- and 1,2-unit content was determined by ^1^H NMR, and the ratio of *cis*-1,4 and *trans*-1,4 unit content of PB was determined by ^13^C NMR. In addition, the ratio of the PS and PB blocks in the copolymer was also determined by ^1^H NMR according to a previously published method. The number-average molecular weights (*M*_n_) and dispersity (*M*_w_/*M*_n_) of the polymers were measured by exclusion chromatography (DAWN EOS) and a size exclusion chromatography-multi-angle laser light scatter instrument (SEC-MALLS; Wyatt Technology). To perform this analysis, the polymer samples were dissolved in tetrahydrofuran with a concentration of 5.0 mg ml^−1^, and the eluent flow rate was 0.5 ml min^−1^. Catalyst morphology was observed using a Zeiss Sigma 300 scanning electron microscope. X-ray photoelectron spectroscopy (XPS) was performed to measure the composition of the α-diimine nickel and nickel (II) naphthenate complexes with a Thermo Scientific ESCALAB Xi+. Differential scanning calorimetry (DSC) curves were collected on a METTLER TOLEDO DSC3 instrument. A 10 mg sample was scanned at a scan rate of 5°C min^−1^ from −150°C to 150°C or 50°C to 300°C. Nyquist plots and potentiodynamic polarization curves were obtained with a ParStat 3000A electrochemical workstation (Princeton, USA). A three-electrode cell was used, and the working, counter, and reference electrodes were samples coated on a stainless copper substrate (1 cm^2^), a platinum plate and saturated calomel, respectively. The electrolyte was 3.5 wt % sodium chloride solution. Electrochemical tests were carried out on the samples in their original states for the blank and samples in the frequency range of 10^−2^ to 10^5^ Hz and the potential range of −0.5 to 1.5 V (1 mV S^−1^), using the polymer coating on the copper matrix as the working electrode, the platinum sheet as the counter electrode, and the saturated calomel as the reference electrode.

## 3. Results and discussion

### 3.1. Synthesis of α-diimine ligand and α-diimine nickel catalysts

**Figure 1.**
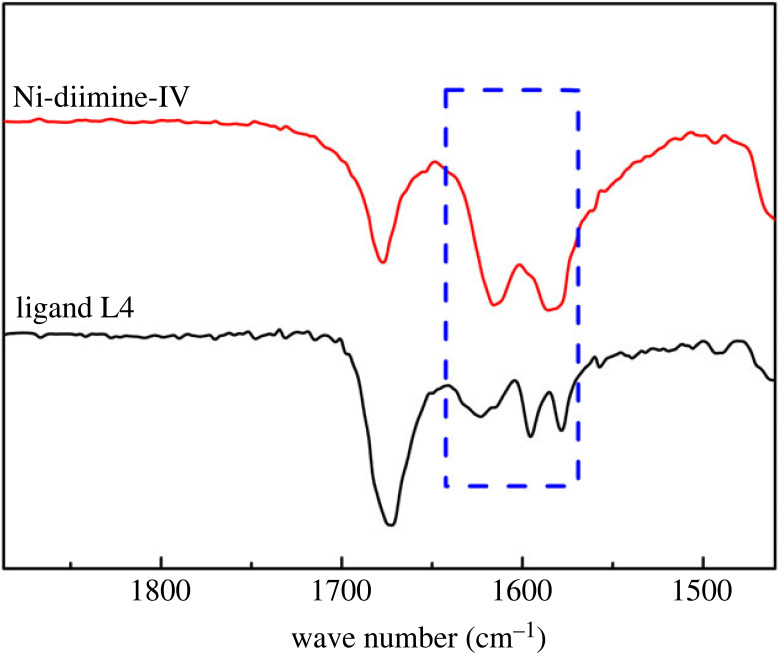
FT-IR of α-diimine ligand L4 and Ni-diimine-IV.

The α-diimine ligands were prepared by a ketoamine condensation reaction, as shown in scheme 1*a*. The FT-IR and ^1^H NMR spectra of α-diimine ligands L1, L2, L3 and L4 are shown in the electronic supplementary material, figures S1–S8. The α-diimine nickel catalysts were prepared using the α-diimine ligands and nickel naphthenate, as shown in scheme 1*b*. The FT-IR spectra of ligand L4 and α-diimine nickel catalyst Ni-diimine-IV are shown in figure 1. In the FT-IR spectrum of L4, a C = N stretching vibration absorption peak is observed near 1624 cm^−1^. The FT-IR spectrum of the catalyst shows C = N stretching vibrations in the low-frequency direction [22,23]. This shows the coordination between the nitrogen atom in the ligand and the central metal ion, indicating the formation of the α-diimine catalyst.

**Figure 2.**
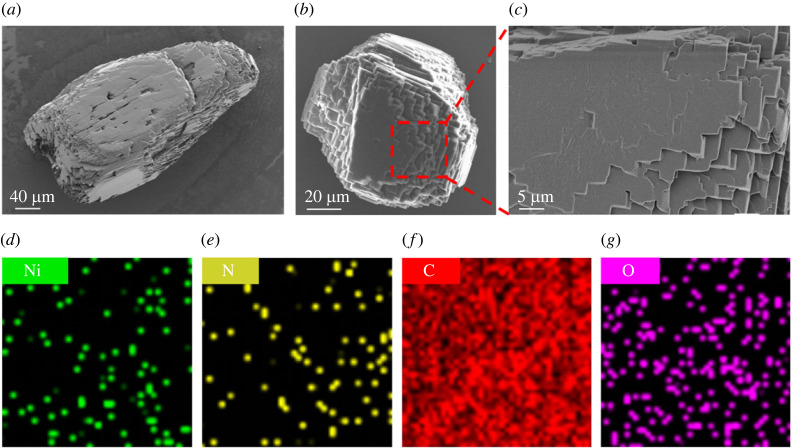
(*a*) and (*b*) SEM image of the Ni-diimine-IV crystal morphology; (*c*) local magnification image of (*b*); (*d*), (*e*), (*f*), (*g*) distribution of Ni, N, C and O elements in the selected area of (*c*).

The morphology structure of the Ni-diamine-IV nickel catalysts was observed by scanning electron microscopy (SEM). As shown in figure 2*a,b*, the catalyst is a monodisperse irregular particle, approximately 500 µm in length and 140 µm in width. It can be seen from figure 2*c*, the Ni-diamine-IV catalyst is lamellar in arrangement, which is owing to the π-π stacking interactions between the benzene rings in the catalysts structure, and the forming stable π-π coplanar aggregates allows the catalyst crystals to grow in a layer growth pattern [24]. In addition, the catalyst was analysed using SEM-energy dispersive spectroscopy, and four elements had been detected, which, respectively, were Ni, N, C and O (figure 2*d–g*). The results also demonstrated that the Ni-diamine-IV catalysts were prepared successfully.

XPS was used to further study the composition and structure of the α-diimine nickel catalyst as shown in the electronic supplementary material, figure S9(a). C, N, O and Ni signals were detected at 284.8, 398.7, 531.1 and 856.1 eV in the full survey spectrum of the α-diimine nickel catalyst. The high-resolution Ni 2p spectrum (electronic supplementary material, figure S9(b)) is divided into four peaks. The satellite peaks are located at 861.2 and 879.1 eV, and two strong peaks corresponding to Ni 2p_3/2_ and Ni 2p_1/2_ are observed at binding energies of 856.3 and 873.9 eV, respectively. These peaks correspond to Ni^2+^. These XPS results further demonstrate the coordination of the α-diimine ligand with nickel [25].

### 3.2. Synthesis of styrene-butadiene di-block copolymer using α-diimine ligand nickel catalysts

#### 3.2.1. Synthesis of styrene-butadiene di-block copolymer using α-diimine nickel catalysts

Owing to the different polymerization activities of styrene and butadiene monomers, the traditional Zigler-Natta nickel system catalyst has high catalytic activity and spatial stereoselectivity for 1,3-butadiene but low polymerization activity for styrene. Thus, it is difficult to obtain block copolymers with high styrene content. To solve this problem, Liu *et al*. [26] used *n*-butyllithium to initiate styrene and obtain the macromolecular alkylation reagent PSLi, which was then combined with nickel naphthenate to achieve copolymerization with butadiene. However, the St block of the PS-*b*-PB obtained by this method presented atactic sequence, with extremely low stereoselectivity. Therefore, in this work, α-diimine and nickel naphthenate were complexed to obtain the α-diimine nickel catalyst, which was combined with Al(*i*-Bu)_3_/BF_3_*Et_2_O/PPh_3_ to achieve the highly efficient polymerization of styrene with highly spatially stereoselective butadiene. Finally, PS-*b*-PB block copolymer was obtained.

Ni-diimine-IV was aged with Al(*i*-Bu)_3_, BF_3_*Et_2_O, and PPh_3_ to create the complex catalyst system. This system was used to initiate the copolymerization of styrene and butadiene. Although the α-diimine nickel catalyst has certain catalytic activity for both styrene and butadiene monomers, its catalytic activity for butadiene is obviously higher than that for styrene. Therefore, the polymerization of styrene is carried out first in this work, and the catalytic active centre after homopolymerization of styrene still maintains a high catalytic activity for the second monomer butadiene, as shown in table 1. The Ni-diimine-IV catalyst system exhibited high catalytic activity and strong stereoselectivity to both monomers. Therefore, the resulting PS-*b*-PB polymer with a distinguished *cis*-1,4 structure unit polybutadiene (*cis*-1,4 > 95%) and high isotactic-selective PS block (*mmmm* > 65%) was obtained.

**Figure 3.**
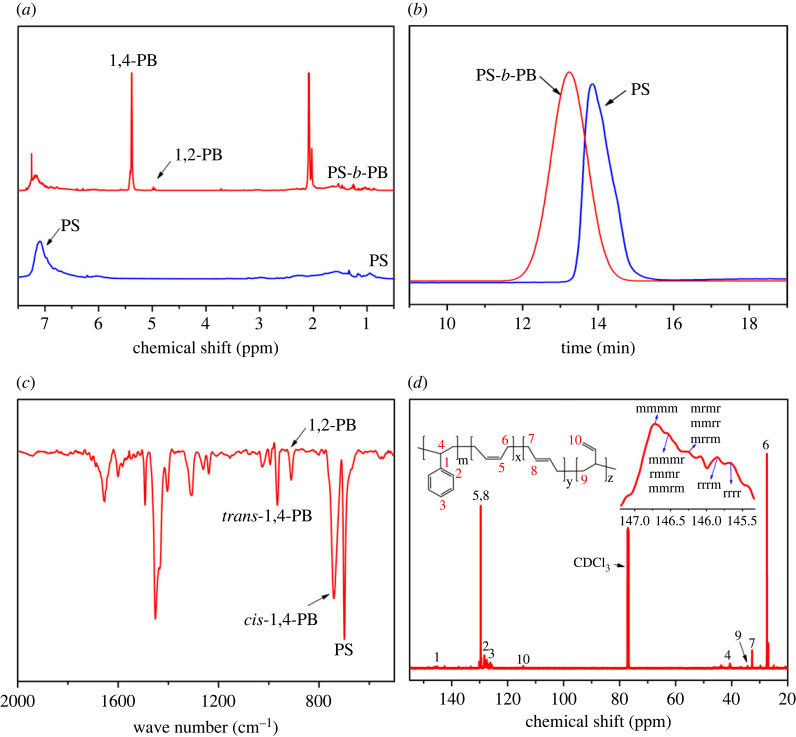
(*a*) ^1^H NMR spectra, (*b*) SEC curve of the polymers in each polymerization steps of PS-*b*-PB, (*c*) FT-IR and (*d*) ^13^C NMR spectrum of PS-*b*-PB via Ni-diimine-IV catalysts.

The ^1^H NMR spectrum, SEC curve, FT-IR spectrum and ^13^C NMR spectrum of the di-block copolymer synthesized with the Ni-diimine-IV catalyst are shown in figure 3. As shown in figure 3*a*, the chemical shifts of the polymer between 6.25 and 7.25 ppm correspond to the benzene rings of the PS block, and the chemical shift peaks at 5.40 ppm and 5.05 ppm correspond to the 1,4- and 1,2- microstructure peaks of the PB block [27,28]. Integrating the chemical shift peaks of the ^1^H NMR spectrum shows that the styrene content in the polymer chains is 29.9% [19,29]. Figure 3*b* shows the step-by-step SEC curves of PS and PS-*b*-PB . The *M*_n_ of the product obtained after styrene polymerization is only 4.3 × 10^4^ g mol^−1^. By contrast, the *M*_n_ of PS-*b*-PB (obtained after the addition of butadiene monomer) is higher and a single peak is maintained, demonstrating that PS and PB homopolymers do not exist in the PS-*b*-PB block copolymer.

The FT-IR spectrum in figure 3*c* shows absorption peaks at 699 cm^−1^ and 1493 cm^−1^ that correspond to the out-of-plane deformation vibration and skeleton vibration of the protons on the benzene ring in the PS block of the polymer. The absorption peaks at 740 cm^−1^, 966 cm^−1^ and 911 cm^−1^, respectively, correspond to the out-of-plane protons in the *cis*-1,4, *trans*-1,4, and 1,2- structural units of the PB block of the polymer. These results show that the PS-*b*-PB copolymer contains high *cis*-1,4 unit content (*cis*-1,4 > 95%) [19]. The ^13^C NMR spectrum in figure 3*d* shows chemical shifts at 27.4 ppm, 32.6 ppm, 34.2 ppm and 144.9–146.4 ppm corresponding to *cis*-1,4, *trans*-1,4, 1,2-, and PS microstructure, respectively [26,30,31]. These results demonstrate that the PS-*b*-PB copolymer is *cis*-1,4-regulated and isotactic-selective (*mmmm* > 65%), which is consistent with the FT-IR results.

#### 3.2.2. Steric volume effects of α-diimine nickel catalysts

α-diimine nickel catalysts with different steric volumes were synthesized by the same process to catalyse the block copolymerization of styrene and butadiene. As shown in table 2, with increasing α-diimine nickel catalyst steric volume, the catalytic activity towards the two monomers improves. At the same time, the *cis*-1,4 unit content stereoselectivity in the PB block and the isotacticity of the PS block significantly increase with increasing steric volume. Compared with the methyl group, the rigid benzene ring structure in the Ni-diimine-III and Ni-diimine-IV catalysts increases their axial steric hindrance and inhibits the rotation of the C_Ar_-N bond to a certain extent. Thus, the stability and activity of the catalytic active centre formed by these catalysts are improved [32]. Meanwhile, the steric hindrance of the substituent group on the N-aryl group improves the molecular weight and the stereoselectivity of the resulting PS-*b*-PB copolymer [33,34]. Therefore, the di-block copolymer prepared with the Ni-diimine-IV catalyst achieves distinguished stereoselectivity with higher *cis*-1,4 unit content in the PB block and higher isotacticity in the PS block.

**Table 2.** Synthesis of PS-*b*-PBs via four different Ni-diimine catalysts^a^.

entry	catalyst	conv^b^	conv^b^ Bd (%)	St cont^c^	*M*_n_^d^ × 10^4^ g mol^−1^	*M*_w_/*M*_n_^d^	*cis*-1,4^e^	*mmmm*^f^
		St (%)		(%)			(%)	(%)
1	Ni-diimine-I	22.8	36.5	19.4	5.0	3.36	70.3	19.2
2	Ni-diimine-II	45.3	53.2	24.5	7.5	2.35	80.5	24.6
3	Ni-diimine-III	60.5	68.3	28.3	8.1	2.87	90.3	53.6
4	Ni-diimine-IV	96.5	82.1	29.9	13.8	1.59	95.2	65.3

^a^First step, [Ni] = 0.01 mmol, catalyst ratio: [Ni]/[St]/[Al]/[B]/[PPh_3_] = 1 : 25 : 20 : 30 : 1, [St]/[Ni] = 400, second step, [Bd]/[Ni] = 2000.

^b^Conversion of monomer in each steps.

^c^Determined by the ^1^H NMR spectrum.

^d^Measured by SEC-MALLS.

^e^Determined by the ^1^H NMR and ^13^C NMR spectrum.

^f^Determined by ^13^C NMR.

#### 3.2.3. Effect of the monomer feed ratio of St/Ni

As shown in table 3, a series of PS-*b*-PBs with different styrene contents were synthesized by changing the amount of styrene added in the first step of the polymerization, and the actual percentage of the PS component in copolymer could be effectively controlled using this Ni-diimine-IV/Al(i-Bu)_3_/BF_3_*Et_2_O/PPh_3_ catalytic system. Moreover, these block copolymers obtained also have high *cis*-1,4-regulated and isotactic-selectivity.

**Table 3.** Effect of St/Ni ratios in the synthesis of PS-*b*-PB^a^.

entry	St/Ni	Bd/Ni	St cont^b^ (%)	*cis*-1,4^c^ (%)	*mmmm*^d^ (%)
5	100	2000	10.4	93.8	50.1
6	200	2000	21.3	94.1	58.1
7	400	2000	29.9	95.2	65.2
8	800	2000	35.7	94.3	64.9

^a^First step, [Ni] = 0.01 mmol, catalyst ratio: [Ni]/[St]/[Al]/[B]/[PPh_3_] = 1 : 25 : 20 : 30 : 1.

^b^Determined by the ^1^H NMR spectrum.

^c^Determined by the ^1^H NMR and ^13^C NMR spectrum.

^d^Determined by ^13^C NMR.

#### 3.2.4. Proposed mechanism

**Scheme 2.**
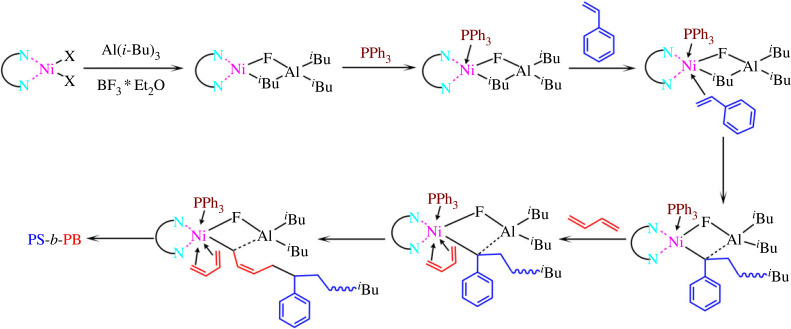
Synthesis mechanism of PB-*b*-PS via Ni-diimine catalysts.

The proposed mechanism of the block polymerization of butadiene and styrene with the α-diimine nickel catalyst is shown in scheme 2, based on the literature reports [35–38]. First, Ni-diimine-IV is reduced by Al(*i*-Bu)_3_ and combines with BF_3_*Et_2_O to form a stable Ni-Al bimetallic active centre. However, the catalytic system has no catalytic activity for styrene and butadiene in this form. When PPh_3_([PPh_3_]/[Ni] = 1) is added, the heteroatom P changes the electron density of the catalytic active centre, which improves the catalytic activity of the system to styrene and butadiene, and also improves the stability of the catalytic active centre. Simultaneously, the steric volume regulation of the catalyst effectively enhances the catalytic activity and stereoselectivity towards the resulting PS-*b*-PB copolymer.

### 3.3. Performance of styrene-butadiene di-block copolymer via Ni-diimine catalysts

#### 3.3.1. Thermal properties

**Figure 4.**
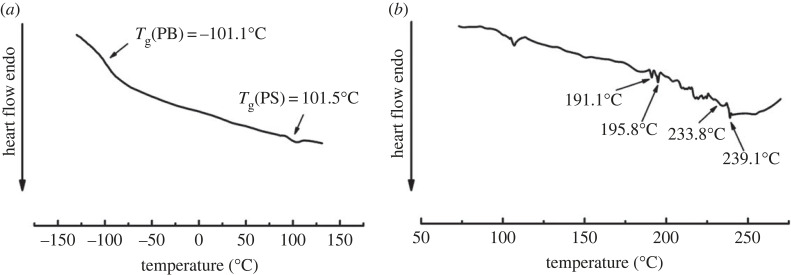
DSC curve of PS-*b*-PB via α-diimine nickel catalysts: (*a*) *T*_g_ of PB blocks and PS blocks; and (*b*) *T*_m_ of PS blocks.

The DSC curve of the PB-*b*-PS sample (entry 4) is shown in figure 4. As shown in figure 4*a*, this di-block copolymer with high *cis*-1,4 unit content (95.2%) has a *T*_g_ of −101.1°C in the low-temperature range, which is very close to the *T_g_* of neodymium-based butadiene rubber [18]. The high-temperature section of the curve shown in figure 4*b* demonstrates that the softening temperature of styrene in the polymer is 101.5°C, and multiple melting peaks appear at 180–240°C. These peaks are close to the isotactic styrene melting peaks reported in the literature [39]. These data further prove that the di-block PB-*b*-PS copolymers containing both high *cis*-1,4 unit content (greater than 95%) and isotactic-rich PS (*mmmm* > 65%) styrene block were successfully synthesized with the α-diimine nickel catalyst.

#### 3.3.2. Electrochemical performance

**Figure 5.**
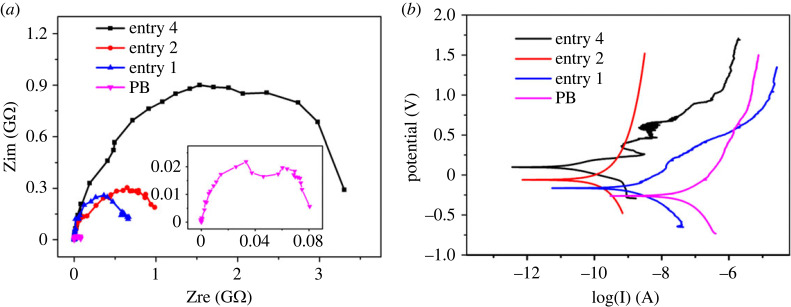
Nyquist plots (*a*) and Tafel curves (*b*) of different polymer coatings.

The corrosion resistance of different polymer coatings was evaluated by Nyquist plot tests and Tafel curves, as shown in figure 5. It shows that as for the PS-*b*-PB coating with high styrene content and high *cis*-1,4 content (entry 4), the PS-*b*-PB coating with low styrene content and high *cis*-1,4 content (entry 2), the PS-*b*-PB coating with low styrene content and low *cis*-1,4 content (entry 1), and the pure PB coating with low *cis*-1,4 content (PB; the synthesis process of PB is reported in the electronic supplementary material, S4), the styrene content and *cis*-1,4 content determine the impedance of the PS-*b*-PB coatings, as shown in figure 5*a* and the electronic supplementary material, table S1. Pure PB (prepared without PS block) has the smallest impedance arc. Higher styrene content and *cis*-1,4 content lead to a larger impedance arc. The pure PB coating has the smallest impedance arc and therefore the lowest impedance value. According to figure 5*b* and the electronic supplementary material, table S2, *I*_corr_ (self-corrosion current) decreases with increasing styrene and *cis*-1,4 content [40,41,42]. This further proves that higher styrene content and *cis*-1,4 content in the PS-*b*-PB polymer coatings lead to better corrosion resistance, which is consistent with the results of the Nyquist plots.

## 4. Conclusion

A series of α-diimine nickel complexes with different steric structures were synthesized. Then, these Ni-diimine complexes were aged with Al(*i*-Bu)_3_, BF_3_*Et_2_O and PPh_3_ to prepare nickel-based catalytic systems, which were successfully used in the copolymerization of styrene and butadiene. The result shows that the Ni-diimine prepared using a ligand with a larger steric volume has higher catalytic activity and can be used to prepare PS-*b*-PB copolymers with better *cis*-1,4 (greater than 95%) and isotactic (*mmmm* > 65%) stereoselectivity. In addition, DSC and electrochemical performance tests show that these block copolymers with *cis*-1,4-regulated PB and isotactic-riched PS have excellent performance in terms of high-temperature and low-temperature resistance as well as corrosion resistance. Therefore, these copolymers are expected to be widely used in some harsh industrial environments.

## Data Availability

The data are also provided in the electronic supplementary material [43].
